# Synthetic Peptides Derived from Bovine Lactoferricin Exhibit Antimicrobial Activity against *E. coli* ATCC 11775, *S. maltophilia* ATCC 13636 and *S. enteritidis* ATCC 13076

**DOI:** 10.3390/molecules22030452

**Published:** 2017-03-12

**Authors:** Nataly De Jesús Huertas Méndez, Yerly Vargas Casanova, Anyelith Katherine Gómez Chimbi, Edith Hernández, Aura Lucia Leal Castro, Javier Mauricio Melo Diaz, Zuly Jenny Rivera Monroy, Javier Eduardo García Castañeda

**Affiliations:** 1 Chemistry Department, Universidad Nacional de Colombia, Bogotá Carrera 45 No 26-85, Building 451, office 409, Bogotá 11321, Colombia; njhuertasm@unal.edu.co (N.D.J.H.M.); jmmelod@unal.edu.co (J.M.M.D.); zjriveram@unal.edu.co (Z.J.R.M.).; 2 Bacteriology Department, Universidad Colegio Mayor de Cundinamarca, Bogotá Calle 28 No. 5B-02, Bogotá 110311; Colombia; yerlycasanova@hotmail.com (Y.V.C.); anyelithgomez_26@hotmail.com (A.K.G.C.); edhernandez@unicolmayor.edu.co (E.H.).; 3 Medicine Faculty, Universidad Nacional de Colombia, Bogotá Carrera 45 No 26-85, Building 471, Bogotá 11321, Colombia; allealc@unal.edu.co; 4 Pharmacy Department, Universidad Nacional de Colombia, Bogotá Carrera 45 No 26-85, Building 450, office 203, Bogotá 11321, Colombia; jaegarciac@unal.edu.co.

**Keywords:** Lactoferricin B, *E. coli*, *S. maltophilia*, *S. enteritidis*, antibacterial activity, synthetic peptides

## Abstract

Linear, dimeric, tetrameric, and cyclic peptides derived from lactoferricin B–containing non-natural amino acids and the RWQWR motif were synthesized, purified, and characterized using RP-HPLC, MALDI-TOF mass spectrometry, and circular dichroism. The antibacterial activity of peptides against *Escherichia coli* ATCC 11775, *Stenotrophomonas maltophilia* ATCC 13636, and *Salmonella enteritidis* ATCC 13076 was evaluated. The minimum inhibitory concentration (MIC) and minimum bactericidal concentration (MBC) were determined. The synthetic bovine lactoferricin exhibited antibacterial activity against *E. coli* ATCC 11775 and *S. enteritidis* ATCC 13076. The dimeric peptide (RRWQWR)_2_K-Ahx exhibited the highest antibacterial activity against the tested bacterial strain. The monomeric, cyclic, tetrameric, and palindromic peptides containing the RWQWR motif exhibited high and specific activity against *E. coli* ATCC 11775. The results suggest that short peptides derived from lactoferricin B could be considered as potential candidates for the development of antibacterial agents against infections caused by *E. coli*.

## 1. Introduction

According to the World Health Organization (WHO), pathogen resistance to conventional antibiotics is considered a global public health problem. The increase of bacterial resistance limits therapeutic options, increasing the length of hospitalization, which raises treatment costs and in some instances can cause death [1,2]. Currently, the majority of infections (urinary, blood, pneumonia, etc.) acquired during hospitalization are caused by resistant strains such as *S. aureus* (MRSA), *E. coli* and *K. pneumoniae*, etc. Bacterial resistance is mainly caused by antibiotic overuse and self-medication [1,2]. *S. maltophilia* is a globally emerging environmental Gram-negative strain that is associated with morbidity and mortality in cancer patients. It causes a wide range of infections. Treatment of *S. maltophilia* infections is difficult, because this pathogen exhibits high levels of intrinsic or acquired resistance to various antimicrobial agents [3,4,5]. *S. Enteritidis* and *S. Typhimurium* are the two most important serotypes of salmonellosis transmitted from animals to humans in most parts of the world. Infections caused by these pathogens have exhibited a gradual decline in susceptibility to traditional antibiotics [6,7]. Over the past few decades, there has been a growing effort to design, develop, and obtain therapeutic agents that do not induce resistance and that could be used for the treatment of infections caused by resistant pathogens. 

Peptide synthesis is considered to be a viable alternative for obtaining designed therapeutic agents against bacterial infections [8,9]. Antimicrobial peptides (AMPs) are part of the innate immune system. More than 800 types of AMPs have been identified in both the plant and the animal kingdoms [10,11]. AMPs are classified as: (i) anionic or cationic peptides containing alpha helices, such as cecropin, magainin, and mellitin; (ii) cationic peptides with numerous cysteine residues and disulphide bridges, such as defensins and protegrins; and (iii) cationic and anionic peptides, products of hydrolyzed proteins such as lactoferricin (Lfcin), which originates from lactoferrin (LF) [10,11]. Bovine lactoferrin (BLF) contains 703 amino acids and is an 80 kDa glycoprotein that is produced on the epithelial cell of the mammalian mucosa. It is mainly found in milk and body fluids [12]. Some biological activities of BLF are: regulator of the immune system, anti-inflammatory, metal absorption and metabolism (particularly iron), procoagulant, granulopoesis, protease inhibitor, anticarcinogenic, and cellular proliferation and differentiation [12,13,14,15]. This protein is part of the innate immune response, exhibiting antimicrobial activity against: parasites, viruses, fungi and Gram-positive and Gram-negative bacteria [12,14,16,17]. It has been suggested that BLF could be relevant for designing and developing therapeutic molecules against infections caused by bacteria, fungi, viruses, and parasites [15,18,19].

Lfcin is located at the N-terminus of BLF. It is obtained from the protein hydrolysis caused by gastric pepsin [16,20]. It has been isolated/described in various mammals, such as humans (LfcinH: ^20^GRRRRSVQWCAVSQPEATKCFQWQRNMRKVRGPPVSCIKRDSPIQCI^66^) and bovines (LfcinB: ^17^FKCRRWQWRMKKLGAPSITCVRRAF^41^), among others [12,21]. LfcinB is a 25-amino-acid peptide that has a high proportion of basic residues, with a net charge of +8, and amphipathic properties [16,20,22]. LfcinB and its derived synthetic peptides exhibited antiviral (HSV-2, HCV, cytomegalovirus, HIV) [23,24], antifungal (*C. albicans*, *C. tropicalis*, *C. neoformans*, *T. mentagrophytes*, *T. rubrum*, *C. uniguttulatus*), antiparasitic (*Clostridium difficile*, *Toxoplasma gondii*, *Eimeria stiedai*), anticarcinogenic (inhibits metastasis in the liver and lungs, cytotoxic activity for leukemia cells, colon carcinoma cell line HT-29, oral squamous carcinoma cell lines, lymphoma cell lines, etc.) and antibacterial activity (*E. coli*, *B. cereus*, *K. pneumoniae*, *B. subtilis*, *C. perfringens*, *P. aeruginosa*, *L. monocytogenes*, *P. vulgaris*, *S. aureus*, *S. bovis*, *Y. enterocolitica*, *S. epidermidis*, *S. haemolyticus*, *S. Hominus*, *Y. pseudotuberculosis*, *S. typhimurium*, *S. Montevideo*, *C. albicans*, *S. enteritidis*, *P. fluorescens*, *S. mutans*, *C. renale*, *E. Jaecalis*, *B. vulgatus*, *B. bifidum*, *B. breve*, *B. longum*) [12,16,19,25,26,27,28,29,30,31]. Studies have suggested that the N-terminal region of lactoferrin binds to the isolated Lipid A, which corresponds to a LPS fragment [12]. It has been suggested that LfcinB interacts with the bacterial plasma membrane through electrostatic interactions between the negatively charged molecules of the bacterial surface and the positively charged residues of the LfcinB. Then hydrophobic residues such as tryptophan interact with the lipid bilayer, inducing membrane disturbance, which leads to membrane instability and permeability and finally rupture [12,21,22,23,24,25]. Moreover, LfcinB has been detected in bacterial cytoplasm, suggesting other mechanisms at the ribosomal, mitochondrial, and nuclear levels [27,28,29,30]. LfcinB exhibits greater antimicrobial activity than BLF, suggesting that this peptide could be responsible for BLF activity [16,20]. Furthermore, LfcinB exhibits greater antimicrobial activity than LfcinH. This has been attributed to the primary sequence of the LfcinB, especially to the RRWQWR motif, which is the minimum described sequence that exhibits antimicrobial activity [31,32,33,34,35]. This motif is amphipathic; the hydrophilic face is positive due to Arg side chains, while the hydrophobic face is due to Trp side chains [12,19,31,32,33,34,35]. It has been reported that short peptides derived from LfcinB exhibit similar or greater antibacterial activity than LfcinB or BLF [19,28,34,36,37,38,39,40,41,42,43].

In the present paper, the antibacterial activity of linear, dimeric, tetrameric, and cyclic peptides derived from LfcinB against *S. maltophilia*, *E. coli* and *S. enteritidis* was evaluated.

## 2. Results and Discussion

**Figure 1:**
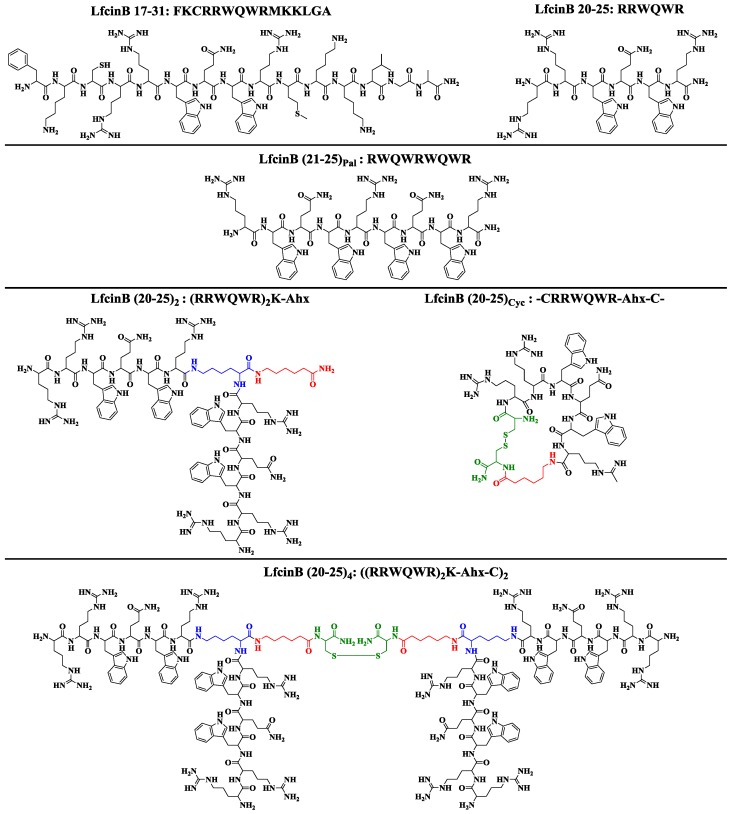
Structure of designed peptides derived from LfcinB.

For this research, the peptides presented in Figure 1 were designed and synthesized through Solid Phase Peptide Synthesis (SPPS), using the manual Fmoc/tBu strategy, specifically: lineal peptides LfcinB (25 residues), LfcinB 17–31 (15 residues), and LfcinB 20–25 (minimal motif, six residues); a dimer (LfcinB (20–25)_2_), a tetramer (LfcinB (20–25)_4_), and a cyclic (LfcinB (20–25)_Cyc_) peptide. Please note that these six peptides contain the minimal motif, and a palindromic sequence (LfcinB (21–25)_Pal_) that contained the RWQWR motif was also included. The crude products were characterized using RP-HPLC and then purified. In all cases, the chromatographic profile of the purified products exhibited the main specie (purity of more than 90%, determined by RP-HPLC). MALDI-TOF-MS analysis showed that synthesized peptides had the expected molecular weight (data not shown).

**Figure 2:**
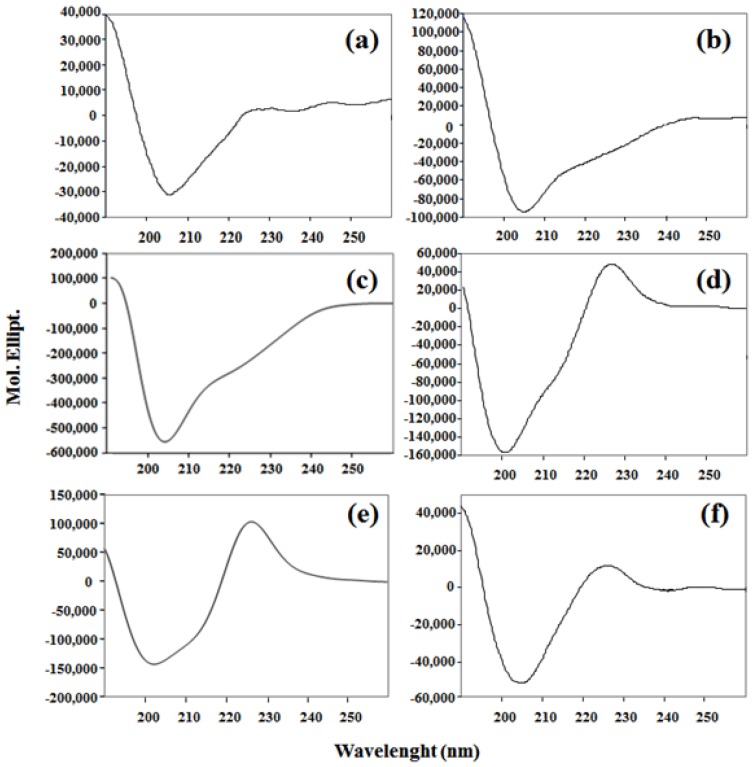
Circular dichroism (CD) spectra of synthetic peptides: (**a**) LfcinB 20–25; (**b**) LfcinB 17–31; (**c**) LfcinB; (**d**) LfcinB (20–25)_4_; (**e**) LfcinB (21–25)_Pal_; and (**f**) Lfcin (20–25)_Cyc_.

In Figure 2, the CD spectra of the synthesized peptides are shown. For lineal peptides LfcinB 20–25 (Panel A), LfcinB 17–31 (Panel B), and LfcinB (Panel C), a similar behavior was found, suggesting that these peptides have a random coil structure; they exhibited a minimum around 206 nm. The CD spectrum of peptide LfcinB 20–25 is similar to that reported for the same sequence, even though the spectra were recorded under different conditions [32]. The CD spectra of tetrameric (Panel D), palindromic (Panel E) and cyclic (Panel F) peptides also exhibited structural features of a random coil, with a minimum between 200 and 206 nm. They exhibited a maximum between 225 and 228 nm. The CD spectra of these peptides differ from the spectrum of peptide LfcinB 20–25, suggesting that polyvalence and the cyclic form affect the secondary structural elements of the motif. The CD spectrum of BLF exhibits a minimum at 238 nm. This is different from the CD spectrum of peptides, which does not have a defined structure. The CD spectrum of BLF exhibits a pattern similar to that previously reported in [44]. However, the minimum is shifted towards 216 nm. This difference is probably caused by the solvents used when recording the spectrum [44]. The results of the CD indicate that it is not possible to establish a structural pattern with the antibacterial activity. This is consistent with the mechanism of action that has been proposed for LfcinB, which is based on the unspecific electrostatic interaction with the negatively charged molecules of the bacterial cell membrane [12]. 

For the evaluation of the antibacterial activity of the synthetic peptides, three bacterial strains were selected: *S. enteritidis*, *E. coli*, and *S. maltophilia*. In the susceptibility assays, it was found that the LfcinB (21–25)_Pal_, LfcinB (20–25)_2_, and LfcinB (20–25)_4_ peptides exhibited the greatest inhibition halos against the three tested strains (11 to 14 mm). The minimum motif LfcinB 20–25 (RRWQWR) was only able to generate an inhibition halo for *S. enteritidis*, while the cyclic peptide LfcinB (20–25)_Cyc_, LfcinB 17–31, LfcinB, and BLF exhibited a small inhibition halo (~9 mm). It is important to indicate that susceptibility assays were carried out at only a one-peptide concentration (200 µg/well). The susceptibility assays are preliminary evidence of antibacterial activity, allowing the visual evaluation of the inhibition of the bacterial growth. The inhibition halo formation could depend on both the antibacterial activity and the physicochemical properties, which can affect peptide diffusion through the agar medium.

For BLF, a Minimum Inhibitory Concentration (MIC) and Minimum Bactericidal Concentration (MBC) larger than 200 µg/mL against the evaluated strains were found (Table 1), implying that high concentrations of the protein are required to achieve antibacterial activity. This suggests that a drug based on BLF could face problems, since it would require high concentrations of BLF. Furthermore, its purification is a long and expensive process. Our results are similar to previous reports, which showed that BLF concentrations greater than 200 µg/mL are required for exhibiting an antibacterial effect on various strains of *E. coli* (0111, L361, ATCC 25922, wild strain, and IID-861) [20,40,42]. On the other hand, synthetic LfcinB exhibited antibacterial activity against *S. enteritidis* and *E. coli* (Table 1, MIC and MBC from 50 to 100 µg/mL). However, for *S. maltophilia*, the MIC/MBC was greater than the maximum tested concentration (200 µg/mL). It is important to notice that peptide LfcinB (20–25)_2_ exhibited greater antibacterial activity than LfcinB against the evaluated bacterial strains. 

The above-mentioned finding is in agreement with previous ones, which indicated that LfcinB exhibits greater antibacterial activity than the native protein [16,20]. Also, our results are in agreement with the antibacterial activity reported for synthetic peptide homologs to peptide LfcinB such as peptide ^17^FKCRRWAQRWRMKKLGAPSITCVRRAF^43^ (27 residues), which exhibits antibacterial activity against *E. coli* ATCC 25922 (MIC = 30 µg/mL) and *E. coli* K88 (MIC= 64 µg/mL) [22,28,37]. Similarly, the shorter peptide KCRRWAQRWRMKKLGAPSITCVR (23 residues) also exhibited antibacterial activity against *E. coli* ATCC 25922 (MIC = 30 µg/mL) [31,41]. LfcinB antibacterial activity (30 to 100 µg/mL) has been reported against *E. coli* ATCC 25922 [30,36,45].

Remarkably, there are some reports that indicate that LfcinB obtained from BLF hydrolysis, using pepsin (LfcinB_hyd_), exhibits greater antibacterial activity against *E. coli* 25922 than chemically synthesized LfcinB. This apparent discrepancy could be related to the method used for determining the MIC [46,47]. The antibacterial activity of LfcinB against *E. coli* IID861 was determined using the microdilution method with Mueller-Hinton broth (MIC = 50 µg/mL) or peptone-based broth (MIC = 6 µg/mL) [36]. LfcinB_hyd_ antibacterial activity has been reported for different strains of *E. coli*, as follows: ATCC 25922 (MIC = 3.3–30 µg/mL), UC6782 (MIC = 10 µg/mL), IID861 (MIC = 50 µg/mL), CL99 1–2 (MIC = 4.13 µg/mL), and K12 UB1005 (MIC = 1.6 µg/mL) [30,39,46,47,48,49,50].

The six shorter synthetic peptides exhibited greater antibacterial activity than the peptide LfcinB and BLF against *E. coli* (Table 1). The best result was obtained with the dimer LfcinB (20–25)_2_, which exhibited a MIC/MBC of 6.2 µg/mL (3 µM); remarkably, the tetramer (5 µM) and the palindromic analog (8 µM) presented a similar activity range to the dimer. For the monomer RRWQWR (minimum motif), the MIC/MBC was 12.5 µg/mL (13 µM). LfcinB 17–31 and the cyclic peptide exhibited values of 13 and 19 µM, respectively. Antibacterial activity against *E. coli* ATCC 25922 has also been reported [39], suggesting that these short peptides exhibit a high degree of antibacterial activity against strains of *E. coli*. This result is interesting because these molecules, being simpler, facilitate the synthetic process and improve yields. Previously we demonstrated that the RWQWR peptide (LfcinB 20–25) presents antibacterial activity against *E. coli* ATCC 25922 [39]. This result is in accordance with Strom et al., who reported that short peptides RWRWRW and RRRWWW exhibit the best antibacterial activity against this bacterial strain [19]. On the other hand, we found that the palindromic sequence LfcinB(20–25)pal exhibit antibacterial activity against two *E. coli* strains, ATCC 11775 and ATCC 25922 (MIC: 12.5 µg/mL and 40 µg/mL respectively). These results are in agreement with previous reports about antibacterial activity against *E. coli* ATCC 25922 of the palindromic sequences: WRWRW (MIC = 15 µg/mL), RWRWR (MIC = 200 µg/mL), WRYRW (MIC = 100 µg/mL) [19]. Please note that palindromic sequences WRWRW and RWQWRWQWR exhibited similar antibacterial activity against this *E. coli* strain, indicating that motifs which alternate Arg and Trp residues enhance the antibacterial activity.

It was found that *S. enteritidis* is susceptible to the short LfcinB analogue peptides. The greatest activity was obtained with the dimer LfcinB (20–25)_2_ (12.5 µg/mL, 6 µM). LfcinB (20–25)_Cyc_ (MIC = 50 µg/mL) and LfcinB (21–25)_Pal_ (MIC= 12.5 µg/mL) are also highlighted in Table 1. We found a greater antibacterial activity against this strain than that reported for LfcinB_hyd_ (MIC = 400 µg/mL) [51], suggesting that shorter synthetic sequences derived from LfcinB could be effective against this bacterium. 

*S. maltophilia* was the bacterial strain least susceptible to the designed short LfcinB analogues. Similarly, the best activity was found for the peptide LfcinB (20–25)_2_ (MIC = 50 µg/mL). Even though the BLF did not exhibit antibacterial activity at the protein concentrations evaluated, previous reports suggest that BLF increases the susceptibility of *S. maltophilia* clinical isolates to Rifampin, suggesting that this effect is due the increased permeability of the cell membrane [52]. The synthetic peptides and the BLF protein did not exhibit hemolytic activity at the tested concentrations (50 to 200 µg/mL).

In summary (Table 1), antibacterial activity against (i) *S. enteritidis* and (ii) *E. coli* was found when short peptides that contain the minimal motif RWQWR were used; (iii) the whole protein (BLF) at the tested concentration did not exhibit antibacterial activity against the tested bacterial strains; (iv) the dimer molecule (Lfcin (20–25)_2_) exhibited activity against *S. maltophilia*, which was the strain least susceptible to the designed peptides. 

This study shows that synthetic peptide analogues of LfcinB exhibit a high degree of antibacterial activity against *E. coli*, even higher than LfcinB and BLF. These findings are in agreement with previous studies, which showed that short peptides derived from LfcinB also exhibited antibacterial activity against strains of *E. coli*. The foregoing suggests that these sequences could exhibit antibacterial activity against *E. coli* and could be considered for the development of new therapeutic agents. Additionally, the present research shows that SPPS is a valuable tool for designing and obtaining new peptide-based therapeutic agents.

## 3. Materials and Methods 

### 3.1. Reagents and Materials

Mueller-Hinton, Agar SPC, Mueller Hinton Broth (MHB), *E. coli* ATCC 11775, *S. maltophilia* ATCC 13636, and *S. enteritidis* ATCC 13076 were obtained from ATCC (Manassas, VA, USA). BLF, *N*,*N*-diisopropylethylamine (DIPEA), triisopropylsilane (TIPS), 1,2-ethanedithiol (EDT), 4-methylpiperidine, pyridine, and ninhydrin were obtained from Sigma-Aldrich (St. Louis, MO, USA). Rink amide resin, Fmoc-amino acids, 6-chloro-1-hydroxy-benzotriazole (6-Cl-HOBt), and *N*,*N*-dicyclohexylcarbodiimide (DCC) were purchased from AAPPTec (Louisville, KY, USA). Methanol, diethyl ether, *N*,*N*-dimethylformamide (DMF), absolute ethanol, dichloromethane (DCM), acetonitrile (ACN), isopropylalcohol (IPA), and trifluoroacetic acid (TFA) were obtained from Honeywell-Burdick & Jackson (Muskegon, MI, USA). All reagents were used without further purification. 

### 3.2. LfcinB-Derived Peptide Synthesis

Peptides were synthesized using manual Solid Phase Peptide Synthesis (SPPS-Fmoc/tBu) [53]. Briefly, Rink amide resin (0.46 meq/g) was used as a solid support. (i) Fmoc group removal was carried out through treatment with 20% 4-methylpiperidine in DMF. (ii) For the coupling reaction, Fmoc-amino acids (0.21 mmol) were pre-activated with DCC/6-Cl-HOBt (0.20/0.21 mmol) in DMF at RT. (iii) Side chain deprotection reactions and peptide separation from the resin were carried out with a cleavage cocktail containing TFA/water/TIPS/EDT (93/2/2.5/2.5 *v*/*v*/*v*). (iv) Crude peptides were precipitated by treatment with cool ethyl ether, dried at RT, and analyzed using RP-HPLC analytical chromatography. 

### 3.3. LfcinB-Derived Peptide Characterization

#### 3.3.1. Analytical Methods

RP-HPLC analysis: 20 µL crude peptide stock solution (1 mg/mL) was analyzed on a C18 column (Eclipse XDB; 3.5 µm; 4.6 × 150 mm), using an Agilent 1200 liquid chromatograph (Omaha, NE, USA). A linear gradient was employed, from 5% to 70% solvent B (0.05% TFA in ACN) in solvent A (0.05% TFA in water). Gradient time was 45 min. A flow rate of 1.0 mL/min, RT, and 210 nm for detection were used. The crude products were purified through solid-phase extraction (SPE) chromatography, a methodology developed in our laboratory. Briefly, Supelclean LC-18 SPE columns were activated and equilibrated, then crude peptides were passed through the column, and the elution was carried out using a gradient of solvent B. Collected fractions were analyzed using RP-HPLC and MS. MALDI-TOF MS analysis was performed on an Ultraflex III TOF-TOF mass spectrometer (Bruker Daltonics, Bremen, Germany) in reflectron mode, using an MTP384 polished steel target (Bruker Daltonics), 2,5-dihydroxybenzoic acid, or sinapinic acid as a matrix, 500 shots with a 25%–30% power laser.

#### 3.3.2. Circular Dichroism (CD)

The CD spectrum for the purified synthetic peptides was recorded following the methodology described in [54]. Peptides (0.2 mM) were dissolved in 2,2,2-trifluoroethanol (30%) aqueous solution and then analyzed in a spectropolarimeter Jasco J-810, between 190 and 260 nm at 25 °C in a quartz cuvette with a 1 cm path length. The result is the average of three scans taken at 20 nm/min with a spectral bandwidth of 1 nm.

### 3.4. LfcinB-Derived Peptide Antibacterial Activity 

#### 3.4.1. Susceptibility Assays

These tests were carried out following the methodology reported in [36]. Briefly, an inoculum aliquot (200 µL; 2 × 106 CFU/mL) was placed on Mueller-Hinton (MH) Agar plates, mixed, and allowed to solidify. Wells were drilled using a punch of 8 mm, and then each hollow was filled with 100 µL of peptide (2000 µg/mL). Incubation for 48 h at 37 °C was then performed. As a non-growth control, ciprofloxacin 1.25 µg/mL was used for all tested strains. Sterile water was used as a growth control.

#### 3.4.2. Antibacterial Activity Assays

The MIC and MBC were determined using a microdilution assay [36]. In brief, bacterial strains were incubated for 18 to 24 h at 37 °C in an MH broth until an optical density of 0.15 to 0.30 (620 nm) was obtained. 90 µL of Mueller-Hinton broth (MHB) were mixed with 90 µL of peptide (440 µg/mL), and using a 96-well microtiter plate peptide, serial dilution (200, 100, 50, 25, 12.5, and 6.2 µg/mL) was performed. 10 µL of inoculum (2 × 10^6^ CFU/mL) was added to each well. Final volume in each well was 100 µL. Then they were incubated for 24 h at 37 °C; and the absorbance at 620 nm was measured using an Asys Expert Plus ELISA reader. To determine the MBC, an aliquot was taken from each well and was spread onto a MHA plate. After 24 h at 37 °C, the CFU/mL was determined. Each of these tests was performed twice.

#### 3.4.3. Hemolytic Activity Assay

Hemolytic activity was determined in accordance with the methodology described in [55]. Red blood cells (RBCs) from a healthy volunteer (O+), were collected in tubes containing heparin and centrifuged (375 g/15 min). The supernatant was discarded, and the cells were washed several times with 0.9% saline solution. Then the cells were re-suspended in PBS (pH 7.4). An aliquot of 100 µL of RBCs (2% hematocrit) was mixed with 100 µL of the peptide stock solution (concentrations from 50 to 200 µg/mL), and then they were incubated for 2 h at 37 °C. The solutions were centrifuged (1150 g/5 min), and the absorbance of each supernatant was measured at 620 nm. This procedure was carried out twice. 

## Figures and Tables

**Table 1:** Antibacterial activity of the designed synthetic peptides.

Antibacterial Activity						
Peptide	*E. coli* ATCC 11775		*S. maltophilia* ATCC 13636		*S. enteritidis* ATCC 13076	
Code	MIC	MBC	MIC	MBC	MIC	MBC
LfcinB *	100 (32.2)	100 (32.2)	>200 (>64.4)	>200 (>64.4)	100 (32.2)	50 (16.1)
**LfcinB 17–31**	25 (12.5)	25 (12.5)	>200 (>100)	>200 (>100)	>200 (>100)	200 (100)
**LfcinB 20–25**	12.5 (12.5)	12.5 (12.5)	>200 (>203)	>200 (>203)	100 (102)	100 (102)
**LfcinB (20–25)_2_**	6.2 (2.8)	6.2 (2.8)	50 (22.4)	50 (22.4)	12.5 (5.6)	12.5 (5.6)
**LfcinB (20–25)_4_**	25 (5.5)	25 (5.5)	>200 (>44)	200 (44)	200 (44)	100 (22)
**LfcinB (20–25)_Cyc_**	25 (21)	25 (21)	200 (84)	200 (84)	50 (42)	50 (42)
**LfcinB 21–25_Pal_**	12.5 (8.5)	12.5 (8.5)	>200 (>136)	200 (136)	25 (17)	25 (17)
**BLF**	>200 (>2.5)	>200 (>2.5)	>200 (>2.5)	>200 (>2.5)	>200 (>2.5)	>200 (>2.5)

MIC and MBC in µg/mL(µM); * LfcinB FKC**RRWQWR**MKKLGAPSITCVRRAE.
